# Effect of Printing Parameters on the Tensile Properties of 3D-Printed Polylactic Acid (PLA) Based on Fused Deposition Modeling

**DOI:** 10.3390/polym13142387

**Published:** 2021-07-20

**Authors:** Ming-Hsien Hsueh, Chao-Jung Lai, Cheng-Feng Chung, Shi-Hao Wang, Wen-Chen Huang, Chieh-Yu Pan, Yu-Shan Zeng, Chia-Hsin Hsieh

**Affiliations:** 1 Department of Industrial Engineering and Management, National Kaohsiung University of Science and Technology, Kaohsiung 807618, Taiwan; mhhsueh@nkust.edu.tw (M.-H.H.); tzen010@gmail.com (Y.-S.Z.); charlie820906@gmail.com (C.-H.H.); 2 Department of Fashion Design and Management, Tainan University of Technology, Tainan 71002, Taiwan; 3 Department of Information Management, National Kaohsiung University of Science and Technology, Kaohsiung 824005, Taiwan; 4 Department and Graduate Institute of Aquaculture, National Kaohsiung University of Science and Technology, Kaohsiung 811213, Taiwan

**Keywords:** FDM, PLA, UV curing, tensile properties, printing angle, raster angle

## Abstract

In order to optimize the efficiency of the Fused deposition modeling (FDM) process, this study used polylactic acid (PLA) material under different parameters (the printing angle and the raster angle) to fabricate specimens and to explore its tensile properties. The effect of the ultraviolet (UV) curing process on PLA materials was also investigated. The results showed that the printing and raster angles have a high impact on the tensile properties of PLA materials. The UV curing process enhanced the brittleness and reduced the elongation of PLA material. Different effects were observed on tensile strength and modulus of specimens printed with different parameters after UV curing. The above results will be a great help for researchers who are working to achieve sustainability of PLA materials and FDM technology.

## 1. Introduction

Three-dimensional printing technology, also known as additive manufacturing (AM), is the process of building objects with complicated and lightweight structures in three dimensions. This technique has grown swiftly over the past decade and is widely applied in different fields, including engineering, construction, medicine, aerospace, etc. [1,2,3]. In the field of traditional manufacturing, producers require a longer circle time and incur higher costs to design and build a mold before starting to manufacture. It is difficult for the traditional method to achieve the requirement of mass customization. The AM technique can build objects with complex structures layer by layer, and it also has the advantages of creating lightweight structures with lower material costs due to its production method [4]. Fused deposition modeling (FDM) is one of the AM building methods that adds thermoplastic materials, including polylactic acid (PLA), polyethylene terephthalate glycol (PETG), polycarbonate (PC) and acrylonitrile butadiene styrene (ABS) with an extrusion nozzle, layer-by-layer, and it is also the most common technique used in AM because it is economical, simple to use, uses multiple materials and has a high processing speed, compared to traditional manufacturing [5,6]. PLA is the most popular material used in FDM because it is biodegradable, environmentally friendly, low cost and highly adaptable to different materials, including carbon, nylon and some other fibers [7,8]. Certain objects made of composites can be irradiated by ultraviolet (UV) light during or after the printing process by a UV curing machine to enhance the combination of the printed materials [9,10]. Therefore, many printing parameters of an FDM 3D printer and the UV curing process can be adjusted which will significantly affect the mechanical properties and manufacturing efficiency of the printed product. The parameters of a 3D printer include the nozzle temperature, the platform temperature, the process speed, the feed rate of the material, the build orientation, the raster angle, the type of infill structure, the infill rate, the layer thickness, the size of the air gap, etc. The parameters of the UV curing process include the wavelength of the UV light, the length of irradiated time and the energy intensity.

The main purpose of this research is to find the optimal method of FDM 3D-printed PLA samples by using certain printing angles of three different types (with X, Y and Z orientations) and five different raster angles of flat orientation specimens. The differences in the tensile properties, with and without the UV curing process of the specimens, were also investigated in this research. The developed method can be applied to optimize the mechanical properties of an FDM-printed composite.

## 2. Literature Review

A large number of scholars have conducted research on analyzing the influence of printing parameters (the infill pattern type, building orientation, raster angle and printing angle) on the mechanical properties of FDM. Jual et al. [11] explored the effect of different infill patterns and infill rates on PLA and concluded that the filling pattern greatly influenced the mechanical properties of the parts with a low infill rate, and that the rectilinear infill type possessed the optimal tensile strength. Wang et al. [12] explored the mechanical properties of the FDM process under different process parameters, including the printing angle, layer thickness, the fill rate and the nozzle temperature of PLA by using uni-axial tensile tests and a dynamic mechanic analysis. Yao et al. and Ye et al. [13,14] established a theoretical model for predicting the failure strength, separation angle and creep deformation of PLA material under a tensile load. The predicted capacity was affirmed by the experimental data and the results indicated that an increased printing angle and a decreased layer thickness can improve the tensile strength of PLA significantly. Naveed [15] investigated the tensile properties of PLA at different raster angles and examined the failure mode to identify the best raster orientation of the layers in the FDM process. Liu et al. [9] fabricated PLA/poly(ε-caprolactone) (PCL) blends and explored the shape recovery performance at three different raster angles: 45/−45°, 30/−60° and 0/90°. Their results indicated that the symmetrical structure of 45/−45° specimens distributed the uni-axial stretching and caused an optimal performance. Bakir et al. [16] explored recycled polyethylene terephthalate (rPET) filament print specimens at three raster angles: parallel (0°), diagonal (45°) and perpendicular (90°), and two build orientations, vertical and horizontal. The result indicated that the raster angle does not have a significant influence on the mechanical behavior of vertical specimens, but the parallel specimens still show a higher ultimate tensile strength (UTS). Sedighi et al. and Cerda-Avila [17,18] both researched the mechanical properties of PLA material in different inner structures that resulted from different printing angles and build orientations. Similar results indicated that the filament arrangement in the specimens that was more parallel to the direction of the tensile load resulted in a higher tensile strength.

Many papers have been written about the assessment of the mechanical characteristics of materials cured by UV irradiation in different composites. Lee et al. [19] improved the mechanical properties of PLA by adding small amounts of a hexafunctional acrylic monomer and a photoinitiator. This study explored the UV curing behavior by using FTIR-ATR spectroscopy and gel fraction determination. The tensile strength, with different hexafunctional acrylic monomer contents and UV doses, was also investigated. The result showed that the mechanical properties of UV-induced, chemically treated PLA improved significantly, compared to neat PLA, due to stronger cross-linking. Ming et al. [20] used a UV-assisted FDM method with a dual-curing process for fabricating continuous fiber-reinforced thermosetting polymer composites (CFRTPCs). This research used UV irradiation to activate the photoinitiator and photosensitizer in order to precure the extruded material, and concluded that the combination of UV irradiation and post-heat treatment offers effective dual-cure solutions for 3D printing, and that it could be a reliable fabrication method for the development of thermosetting composite systems.

To summarize, there is a paucity of research on the effects of the mechanical behavior of samples printed at certain printing angles on the X, Y and Z axes or orientations. Therefore, this paper aims to explore the differences between neat PLA tensile specimens printed at different angles of inclination, based on three axes, in the FDM process. This research also focuses on determining the differences between the mechanical properties of the PLA samples before and after the UV curing process.

## 3. Experimental

The purpose of this research is to analyze the tensile properties of PLA parts fabricated by FDM. An X1E 3D printer (Infinity3DP, Kaohsiung, Taiwan) fabricated the tensile specimens with a nozzle size of 0.4 mm, a nozzle and platform temperature of 205 °C and 60 °C, respectively, and the infill density was set at 10%. The parameters of printing of the filaments that were adopted to prepare the FDM specimens are shown in Table 1. The PLA material was the Snow-white PLA produced by the MIN-YAU Company (Taipei, Taiwan) with a diameter of 1.75 mm, and the main structure and the support structure were printed by using the same nozzle. The main purpose of this research is to study the effects of certain parameters, such as the printing angle, the build orientation and the raster angle. The effects on the mechanical properties of the UV curing process are also investigated.

**Figure 1:**
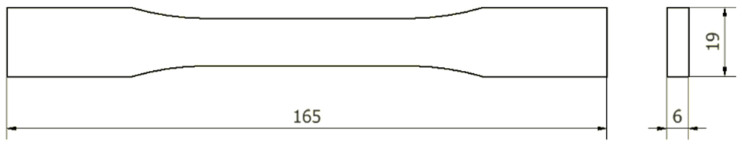
Specifications of the tensile test specimens.

**Figure 2:**
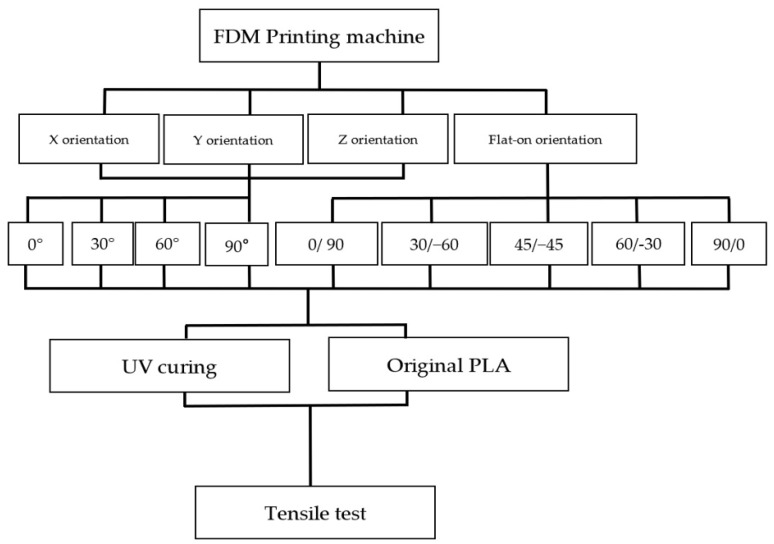
Schematic diagrams of the experimental process.

The tensile measurement specimens were drawn as a digital model and imported as an STL format file by Inventor software, according to the ASTM D638 standard testing method shown in Figure 1. Cura 3.4 software was used to adjust the printing angle (0°, 30°, 60°, 90°) of three different build orientations (the X, Y and Z axis). The specimens were named according to the printed orientation and the angle of tilt to the platform, such as X30 and Y90. The schematic diagrams are shown in Figure 2 and Table 2. It should be noted that the specimens with 90° on the *X* axis and 90° on *Z* axis and 90° on the Y axis and 0° on the *Z* axis were totally identical in the printing process, and therefore they were tested with the same specimens. The STL file was sent to KISSlicer software to slice the model and transform it into a G-code format for the printer to build specimens. The type of raster angle specimens printed in a flat orientation was also defined in this software and named 0°/−90°, 30°/−60°, 45°/−45°, 60°/−30° and 90°/0°, respectively. The scheme of the raster angle type specimens is also depicted in Table 2. For each condition, five specimens were printed and prepared for testing. To explore the effect of the UV curing process, two sets of identical specimens were printed and one of the sets was prepared for irradiation by UV light.

After the 3D printer constructed the complete model, the specimens were immediately put into a UV curing machine for the UV light to cure them. The Multicure 180 UV curing machine (XYZ PRINTING, New Taipei Taiwan) was used. A wavelength of 365 nm and a power intensity of 60% were selected, and the specimens were put in the machine and irradiated by the UV light in batches. The rotational speed of the turntable was 1 revolution per minute. Then, the specimens were placed in a humidity-controlled box to keep them dry and to prepare them for the tensile test.

The uni-axial tensile test was conducted according to ASTMD638 and performed on a YM-H51 (Yang Yi Technology, Tainan, Taiwan) universal test machine, with the specimens being tested at a speed of 5 mm/min and a force of 100N. The built-in software collected the test data to obtain the value of the tensile strength, the strain and Young’s modulus.

## 4. Results and Discussion

**Figure 3:**
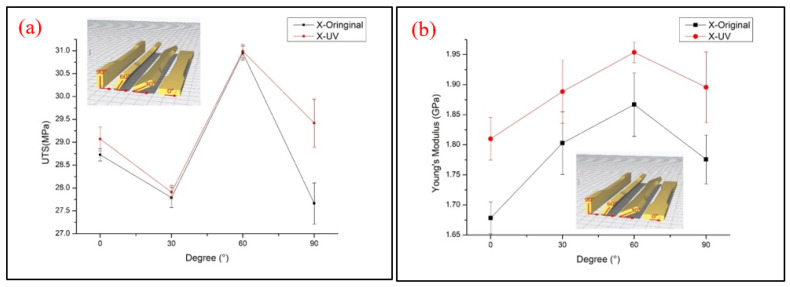
Tensile test result of (**a**) UTS and (**b**) Young’s modulus of the X-type samples.

**Figure 4:**
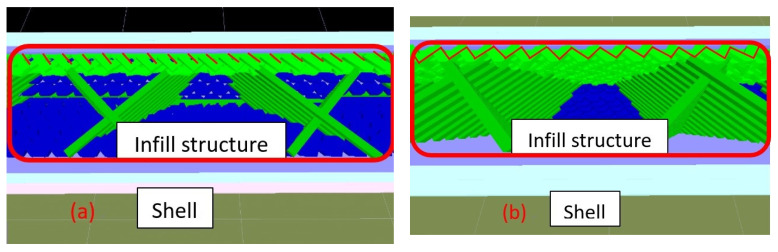
The infill structure of the (**a**) X30° and (**b**) X60° samples.

**Figure 5:**
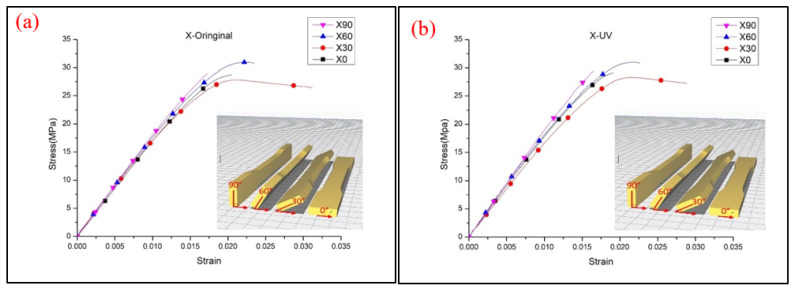
The stress–strain curve of the X-type specimens (**a**) original and (**b**) UV.

**Figure 6:**
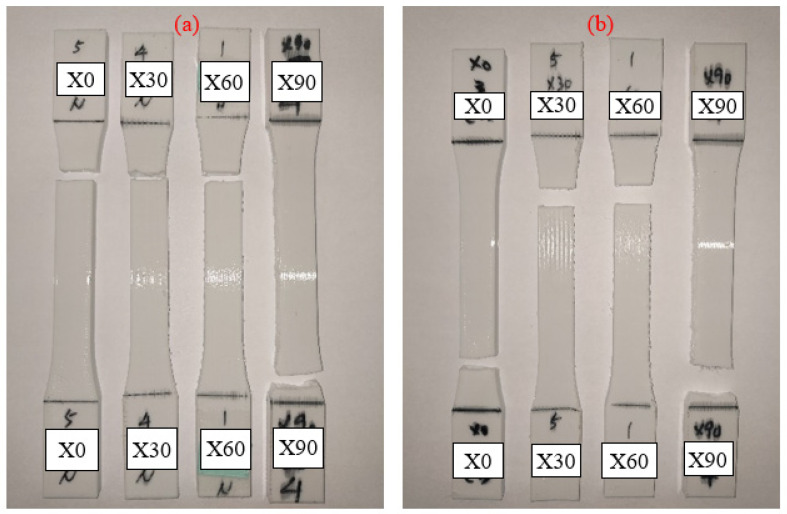
The X-type fracture specimens (**a**) original and (**b**) UV.

For each type of parameter of the specimens, five identical specimens were produced and tested. The average values of triplicates of the individual parameters (UTS, Young’s modulus and stress–strain curve) were selected. The specimens with 90° in the *X* axis and 0° in the *Z* axis, 90° in the *Y* axis and 0° in the *Z* axis and 0° in the *X* axis and 0° in the *Y* axis were tested and the same test data were obtained. Figure 3 demonstrates the UTS values and Young’s modulus at each printing angle. It seems that the printing angle is a critical parameter in the FDM process and that it has a significant impact on the tensile behavior of PLA material. With or without the UV process, the X60° specimen shows the highest tensile strength. The result is not only caused by the fibers being parallel to the tensile load direction on the interface of the specimens, but also by the concentrated structure attached to the inner shell, which are both caused by the tilt angle of the specimens. This kind of structure appears in the X30° and X60° printed specimens; from the slice software, as shown and marked with a red line in Figure 4, the concentrated structure of X60° was a continuous structure, which allowed the samples to resist the applied tensile; however, the X30° specimens did not show a continuous structure and might have resulted in the lower UTS value of X30° [21]. Another possible reason for the zigzag trend might have been that the low infill density could not present the properties of PLA material. The defect and the cave structure during the FDM process may have caused the unstable mechanical characteristic of specimens [15]. The results of the specimens after the UV curing process show the same trends as the original specimens. It can be seen that the UTS value increased slightly after UV curing, but that there is a larger margin in the X90° samples. The stronger mechanical properties are caused by the further enhancement of the cross-linking of PLA filaments [8]. The stress–strain curve of the X-type specimens indicates that the UV curing process decreased the elongation of PLA material, which is caused by the enhancement of the functionality of the acrylates and results in them being less flexible [22]. The stress–strain curve is illustrated in Figure 5. To better determine the fracture properties, two failure modes are defined, namely, the interlayer and in-layer failure modes. The fracture specimens in Figure 6 all demonstrate the in-layer failure mode and elastic–plastic material behavior [15].

**Figure 7:**
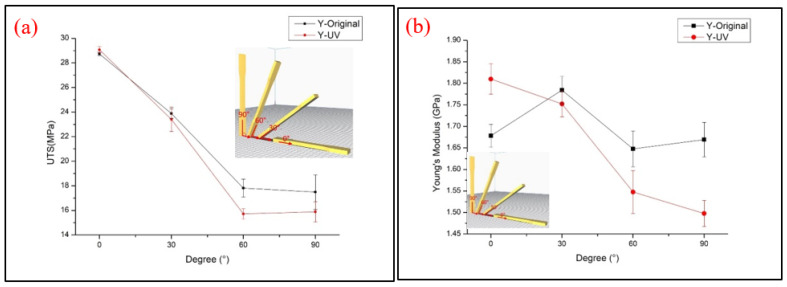
Tensile test results of (**a**) UTS and (**b**) Young’s modulus of the Y-type samples.

**Figure 8:**
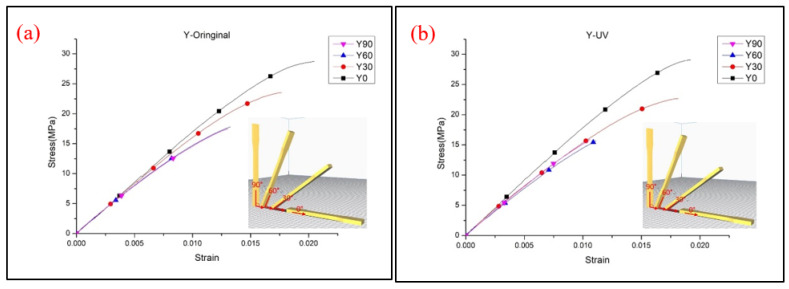
Stress–strain curve of the Y-type specimens (**a**) original and (**b**) UV.

**Figure 9:**
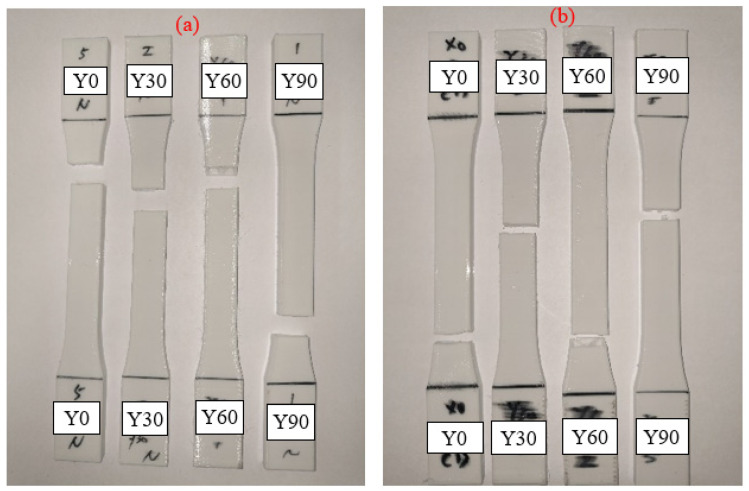
Image of Y-type fracture specimens (**a**) original and (**b**) UV.

The Y-type specimens, the tensile strength and Young’s modulus results are demonstrated in Figure 7. Most specimens had weaker properties, and the tensile strength decreased with the increasing printing angle, which presented a different result from other types of specimens. The Y0° specimens show the strongest UTS value of specimens for the Y-type orientation. The schematic shows that only Y0° specimens were pasted onto the platform and that the building orientation with the fibers parallel to the tensile load direction caused a higher tensile strength. With the increasing degree of printing angle, the included angle between the filaments and tensile load direction is closer to 90°. When the specimens were printed over 0°, the interlayer fracture type appeared on the specimens and affected the combination between layers. The impact rose with the increasing printing angle, which led to the downtrend of UTS and Young’s modulus value [2]. The trend of the results is similar to those in Wang et al.’s research [12]. Figure 8 illustrates the stress–strain curve of the Y-type specimens. The rest of the Y30°, Y60° and Y90° specimens were all produced with a vertical layer to the tensile load direction. The UV curing process embrittled the material, which led to the easy failure of the cured specimen during the tensile load. A significant reduction in elongation could also be seen after the UV curing process. The interlayer failure mode can be seen from the image of the tested specimens shown in Figure 9. It should be noted that a lower Young’s modulus is shown in the Y60° and Y90° specimens after the UV curing process. The crossing of the line may be caused by the over-irradiation of UV light in the low infill density of PLA specimens, which embrittles the material and causes the PLA filaments to fracture easily when tensile stress is applied to specimens [23].

**Figure 10:**
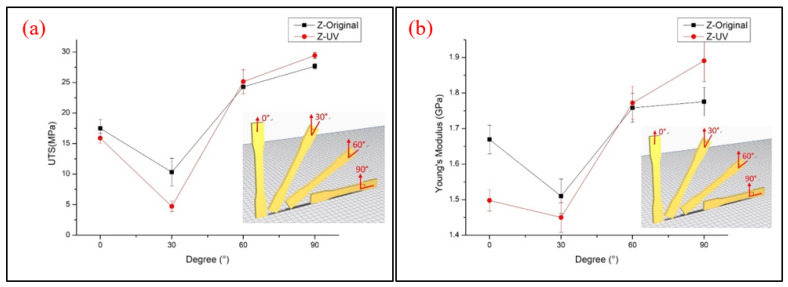
Z-type specimen tensile test results of (**a**) UTS and (**b**) Young’s modulus.

**Figure 11:**
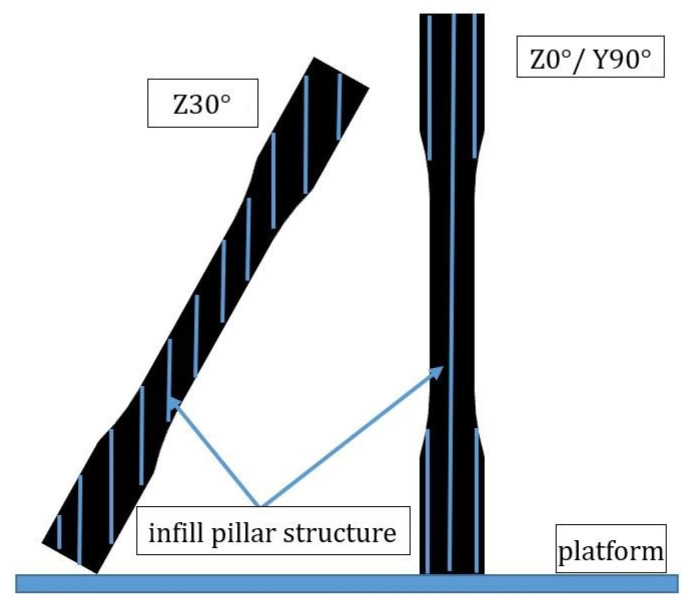
Diagram of infill pillar structure of Z30° and Z0/Y90° specimens.

**Figure 12:**
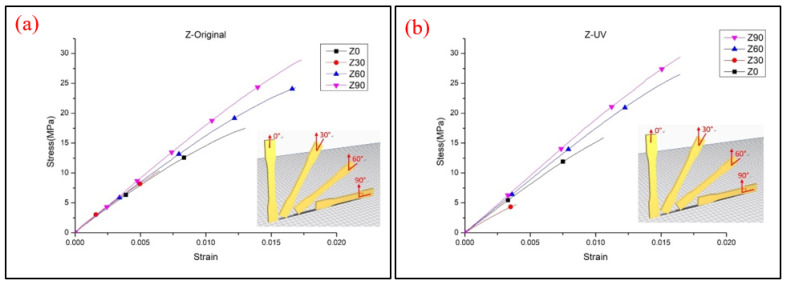
Stress–strain curve of Z-type specimens (**a**) original and (**b**) UV.

**Figure 13:**
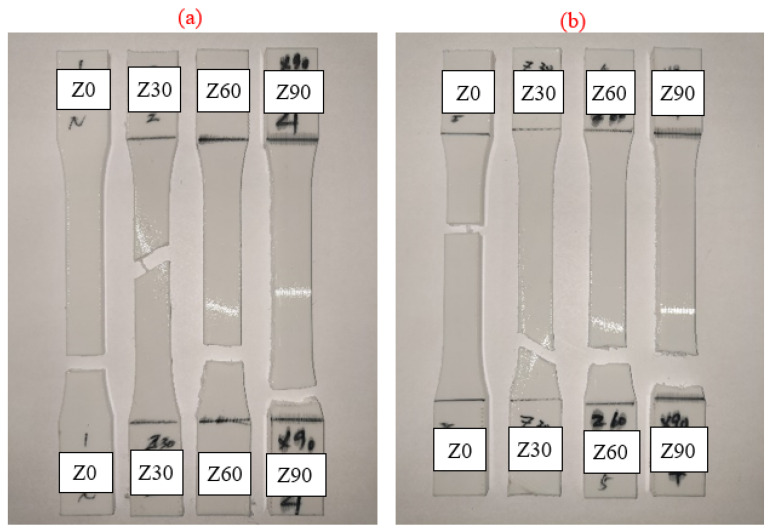
Image of fracture Z-type specimens (**a**) original and (**b**) UV.

The obtained data from the tensile test of the Z-type specimens are shown in Figure 10. Most of the tensile strength and Young’s modulus values are increased with the increasing printing angle, except the Z30° specimens that show a significant reduction, which indicates a different result from previous research [12]. The reason for the poor properties is probably because of low infill density in the inner structure. Figure 11 shows the inner structures of Z30° and Z0° specimens; it can be seen that the infill pillar structures of Z30° are short and dispersed, which results in the specimens not being able to bear the tensile load. This can be compared to the Z0° specimens, in which the longer infill pillar structure provides the specimens with a better ability to resist the tensile load. The trend of Young’s modulus also corresponds to the results of the lower tensile properties, but the rising trend is greatly increased when specimens are printing at 90°, due to the elastic–plastic behavior of the specimens. The short inner infill structure could also be seen in Z60° specimens, but the larger degree of filament orientation of the interface shell provides the material with a better ability to resist the tensile load. The UTS value of Z0° and Z30° specimens decreases after the UV curing process, while others are only slightly enhanced. Figure 12 illustrates the stress–strain curve of Z-type specimens. A significant reduction in elongation can be seen on cured specimens. Due to the vertical direction of patterns to the tensile loading and the brittle properties of the material after the UV curing process, the specimens fracture easily during tensile loading. The fracture specimens are shown in Figure 13. It seems that a printing angle of over 60° demonstrates an in-layer failure mode, and less than 30° presents an interlayer failure mode [13].

**Figure 14:**
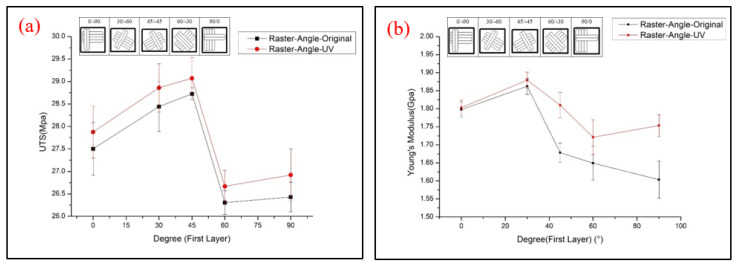
Tensile test results of (**a**) UTS and (**b**) Young’s modulus of flat-type samples.

**Figure 15:**
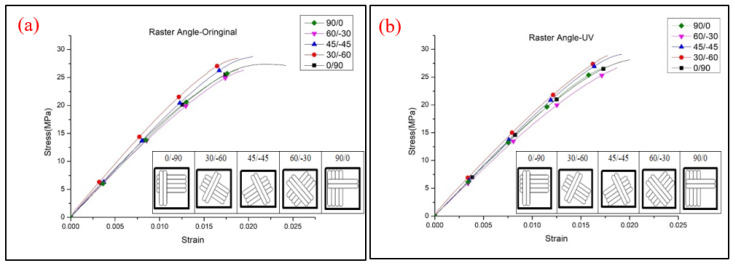
Stress–strain curve of flat-type samples (**a**) original and (**b**) UV.

**Figure 16:**
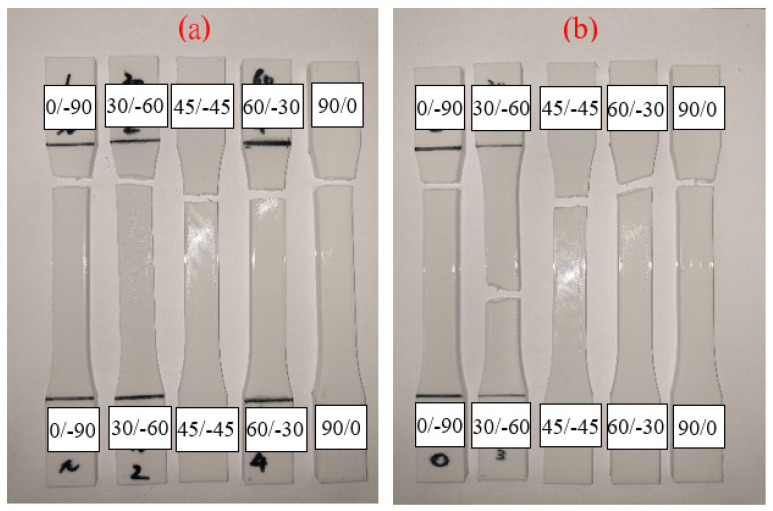
Image of flat-type fracture specimens (**a**) original and (**b**) UV.

The tensile properties of flat-type specimens with different raster angles are shown in Figure 14. The 45/−45° specimens show the highest UTS value and the specimens printed with 60/0° produce the weakest values, which is caused by the fiber direction of the 45/−45° specimens, which could resist and separate the tensile that was applied evenly [9]. The lowest UTS value was probably caused by the longer duration of the process route of the nozzle. The larger angle of the raster pattern results in the nozzle of the specimens with a 60/−30° raster angle moving a longer distance from one layer to another. The filaments of previous layers that are already cooling during manufacturing cause weaker bonding between the layers [24,25,26]. It should be noted that a variation in the raster angle and the UV irradiation affect the tensile strength and elongation of flat-type specimens, which are quite small, especially the 0/90°, of 1.797MPa and 1.803MPa before and after UV curing, respectively, as shown in Figure 15. The reason may be the effect of low infill density on the flat-type specimens, which could not completely present the mechanical behavior of the original PLA. A few mechanical properties increased in the flat-type specimens after the UV curing process. Figure 16 demonstrates an image of the fractured specimens. An interlayer failure mode was observed in the flat-type specimens and demonstrates the elastic–plastic behavior of the material.

## 5. Conclusions

In this study, four types of specimens were produced, and four different printing angles were used on the X, Y, Z orientations of specimens and five different raster angles were used on the flat-type specimens. The effects of the tensile properties, with and without the UV curing process of the PLA-fabricated samples, were investigated to determine the optimal parameters of the FDM printing process. The results indicate that the printing angle has a large impact on the mechanical properties of the FDM printed part, and that the X90°/Z0° specimens among three types of specimens and the 45/−45° of flat-type specimens have the strongest tensile properties. The printing angles of Z30° and Y90°/Z0° are not advised as printing parameters because of the weak bonds between the layers, and the vertical direction of the tensile load causes poor elongation of the parts, which can easily fail. A downtrend with increasing printing angle could be observed on Y-type samples due to the transversal direction of fibers. The effect of the UV curing process on the UTS value and Young’s modulus of elastic–plastic samples, such as the X-type and the flat-type specimens, are slightly enhanced; however, when the filament orientations of specimens are vertical to the tensile loading, such as in the Y30°, Y60° and Y90° samples, the UV curing process further increases the brittleness of the material, which can lead to a reduction in UTS and the tensile modulus. The fracture specimens demonstrate an interlayer failure mode. A significant decrease in elongation could be observed in all the specimen types after being UV irradiated. Table 3 provides a comparison of the tensile results of this paper and previous publications, and it can be seen that solid infill percentage could better respond to the mechanical properties of the original material. Lower infill density could reduce the cost of materials and labor, but may cause unstable mechanical properties of test specimens, Hence, a higher infill density should be selected in further research. The results of this research are expected to contribute to manufacture with FDM.

## Figures and Tables

**Table 1:** Processing parameters of FDM.

Parameter	Value
Nozzle temperature	205 °C
Nozzle diameters	0.4mm
Printing speed	35 mm/s
Infill density	10%
Platform temperature	60 °C
Layer thickness	0.2mm
Filament diameters	1.75mm
Printing axis	X, Y, Z
Printing angle	0°, 30°, 60°, 90°
Raster angle	0°, 30°, 45°, 60°, 90°

**Table 2:** Definition of the tensile test specimens.

Type	Name	Schematic Diagram
X Orientation	X0	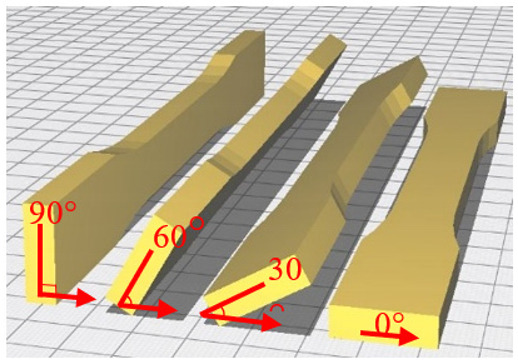
	X30	
	X60	
	X90	
Y Orientation	Y0	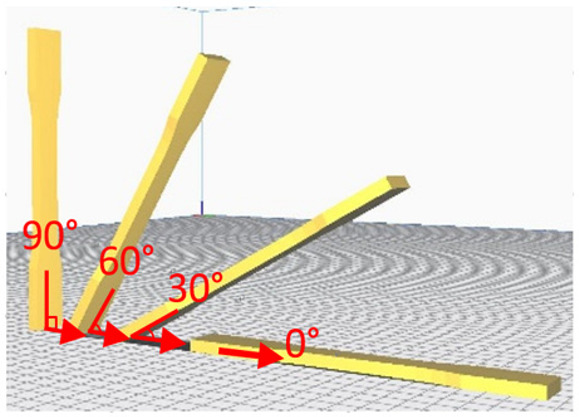
	Y30	
	Y60	
	Y90	
Z Orientation	Z0	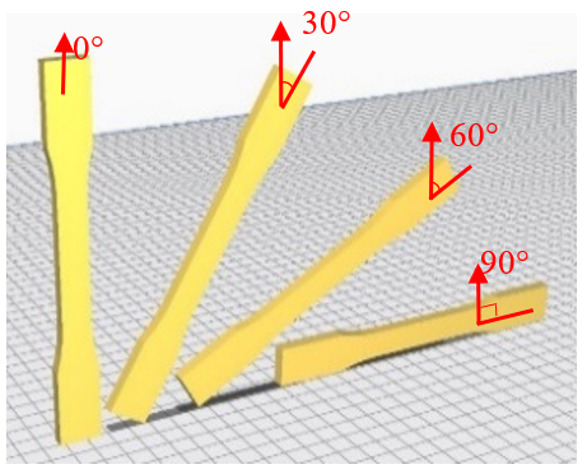
	Z30	
	Z60	
	Z90	
Flat-on orientation with different raster angles		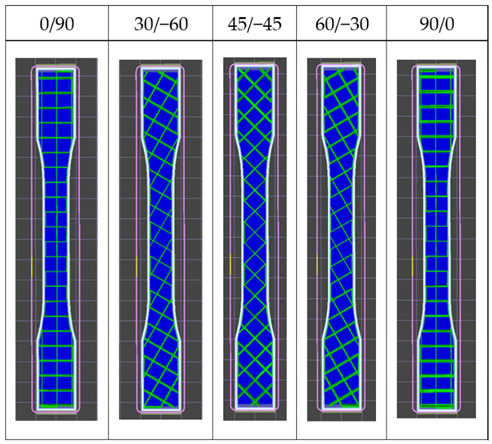

**Table 3:** Comparison of obtained tensile results from previous publications.

**Printing Angle**	**Tensile Strength (MPa)**			**Young’s Modulus (GPa)**		
	**X**	**Y**	**Z**	**X**	**Y**	**Z**
0°	28.7	28.7	17.4	1.678	1.678	1.669
30°	27.7	23.8	10.3	1.802	1.784	1.510
60°	30.9	17.8	24.2	1.866	1.647	1.758
90°	27.6	17.4	27.6	1.775	1.669	1.775
0°	-	-	-	-	-	-
90° [12]	20.0	-	20.0	1.459	-	1.459
0°	75.2	75.2	28.8	3.867	3.867	4.011
90° [10]	73.2	28.8	73.2	4.042	4.011	4.042
0°	-	-	24.93	-	-	1.35
90° [13]	54.22	24.93	54.22	2.68	1.35	2.68
0°	-	-	20	-	-	-
90° [27]	53	20	53	-	-	-
0°	45	45	8	2.8	2.8	2.1
30°	-	-	35	-	-	2.8
60°	-	-	12.5	-	-	2.2
90° [4]	55	8	55	3.5	2.1	3.5
**Raster Angle**	**Tensile Strength (MPa)**			**Young’s Modulus (GPa)**		
0/90°	27.50			1.7977		
30/−60°	28.44			1.8622		
45/−45°	28.72			1.6783		
60/−30°	26.30			1.6493		
90/0°	26.43			1.6033		
0°	14.03			0.94		
30°	17.11			0.85		
45°	55.45			1.46		
60°	15.27			0.87		
90° [15]	40.33			1.67		
0°	33.3			1.44		
45°	35.8			1.41		
90° [16]	47.7			1.73		
0/90°	51			1.5		
45/−45° [28]	52			1.4		
